# Sensitivity of MRI reports for ligamentous injuries in high-grade knee dislocations: A single-center retrospective analysis of radiology reports and operative findings

**DOI:** 10.1051/sicotj/2025046

**Published:** 2025-08-04

**Authors:** Magd Boutany, David Abdelnour, Alqasim Elnaggar, Eric Schweller, Rahul Vaidya

**Affiliations:** 1 Department of Orthopaedic Surgery, Wayne State University School of Medicine 540 E. Canfield St. Detroit MI 48201 USA; 2 Department of Orthopaedic Surgery, Wayne State University Detroit Medical Center 4201 St Antoine Detroit MI 48201 USA

**Keywords:** Knee dislocations, MRI, Diagnostic accuracy, Schenck classification, Retrospective study

## Abstract

*Introduction*: Knee dislocations, particularly high-grade injuries such as Schenck class KDIV, are complex injuries often resulting from high-energy trauma. While magnetic resonance imaging (MRI) is widely used preoperatively to assess ligamentous damage, its diagnostic accuracy remains uncertain. *Methods*: A retrospective review was conducted on 92 patients who underwent surgery for a knee dislocation at a Level I trauma center over 10 years. Patients who had a preoperative MRI report and intraoperative confirmation of a KDIV injury without a tibial plateau fracture were included, which left 31 patients. MRI sensitivity was determined by comparing radiology reports to operative findings with fluoroscopic examination under anesthesia (EUA) for injuries to the anterior cruciate ligament (ACL), posterior cruciate ligament (PCL), medial collateral ligament (MCL), lateral collateral ligament (LCL), and posterolateral corner (PLC). Postoperative follow-up documents were reviewed for functional outcomes. A one-way analysis of variance (ANOVA) was performed to evaluate differences in sensitivity across ligament types, followed by a Tukey post hoc test for pairwise comparisons. Mean flexion ROM at final follow-up (≥6 months) was compared between the accurate and inaccurate MRI cohorts using an independent *t*-test. *Results*: Only 35.5% of MRI reports fully matched operative findings. MRI sensitivity was 71.0% for the ACL (22/31), 61.3% for the PCL (19/31), 93.5% for the MCL (29/31), 64.5% for the LCL (20/31), and 51.6% for the PLC (16/31). ANOVA revealed that MCL sensitivity was significantly higher than that of the PLC, PCL, and LCL. The difference in mean flexion ROM at final follow-up between accurate and inaccurate MRI cohorts was not statistically significant (*p* = 0.56). *Discussion*: Preoperative MRI radiology reports demonstrated substantial limitations in accurately identifying ligamentous injuries in KDIV knee dislocations, particularly involving the PLC, PCL, and LCL. These findings highlight a gap between radiologic interpretation and surgical findings. Surgeons should interpret MRI reports with caution and incorporate fluoroscopic EUA at the time of surgery to ensure a comprehensive assessment of ligamentous damage.

## Introduction

Knee dislocations are severe injuries that often result from high-energy trauma such as motor vehicle accidents. However, they may also occur from sports-related events or low-velocity mechanisms like falls [1, 2]. Knee dislocations have substantial long-term implications, with many patients being unable to return to sports or forced to change professions due to lasting instability or dysfunction [1]. The Schenck classification system is commonly used to categorize knee dislocations based on the number and combination of torn ligaments, with KDIII, KDIV, and KDV classifications indicating the most extensive ligamentous damage [3].

Radiological imaging plays a crucial role in assessing the severity and extent of injury, and is essential for surgical planning [4, 5]. Pre-reduction anteroposterior (AP) and lateral radiographs help assess the direction of dislocation and identify associated fractures, while post-reduction images confirm alignment [4, 5]. If fractures are observed, a preoperative CT scan may be performed to evaluate the fracture pattern in more detail [4].

Magnetic resonance imaging (MRI) is a valuable, non-invasive radiological modality used to assess soft tissue and ligamentous injuries before surgery [4, 5]. However, its diagnostic accuracy in the setting of knee dislocations remains a matter of controversy. Derby et al. reported that MRI effectively identified injuries to the anterior cruciate ligament (ACL) and posterior cruciate ligament (PCL) but frequently missed injuries to the posterolateral corner (PLC) [6]. Similarly, Li et al. reported that while MRI was effective in identifying specific individual ligament injuries, its overall accuracy diminished when evaluating multiple-ligament knee injuries [7].

Most prior studies have assessed MRI performance through retrospective image reviews conducted by musculoskeletal radiologists [6, 7]. In contrast, surgical decision-making at the time of care typically involves dependence on the radiology report issued contemporaneously by the interpreting radiologist and review of the radiological images by the orthopaedic surgeon prior to surgery [8]. This study aims to evaluate the diagnostic accuracy of preoperative MRI reports in identifying ligamentous injuries in patients who had KDIV knee dislocations, which were confirmed by examination under anesthesia and intraoperative findings.

## Methods

**Figure 1:**
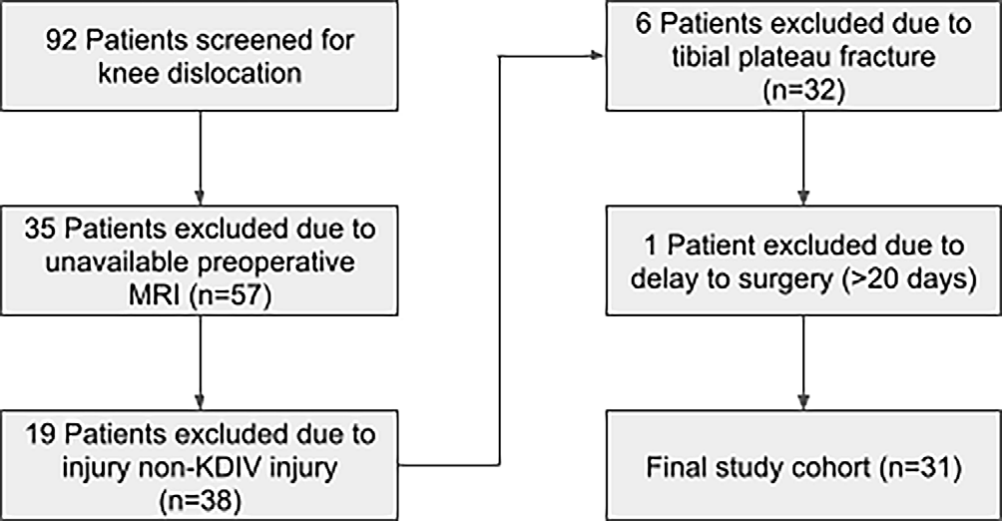
Patient inclusion and exclusion flowchart illustrating the selection of the final study cohort. Beginning with 92 patients screened, exclusions were made for absence of preoperative MRI (*n* = 35), non-KDIV injury classification (*n* = 19), tibial plateau fracture (*n* = 6), and delay to surgery > 20 days (*n* = 1), resulting in 31 patients included in the analysis.

An institutional review board-approved retrospective review was conducted on 92 patients who underwent surgery for a knee dislocation at a Level I trauma center. The inclusion criteria were patients with intraoperative confirmation of a Schenck class KDIV knee dislocation (Table 1), no tibial plateau fracture, and availability of a preoperative MRI with a corresponding radiology report dictated by a fellowship-trained musculoskeletal radiologist within 20 days of surgery. Thirty-one patients met these criteria and were included in the study (Figure 1).

**Table 1:** Summary of the Schenck classification system.

Type	Description
KDI	Two ligaments ruptured, one cruciate and one collateral
KDII	Both cruciate ligaments ruptured
KDIIIM	Both cruciate ligaments ruptured, and MCL ruptured, LCL spared
KDIIIL	Both cruciate ligaments ruptured, and LCL ruptured, MCL spared
KDIV	Both cruciate ligaments ruptured, and both collateral ligaments ruptured
KDV	Fracture Dislocation

Patient demographic information, preoperative MRI reports, OR findings, and postoperative follow-up notes were obtained from the medical record. MRI and OR reports were reviewed retrospectively to assess the accuracy of the MRI reports in identifying injuries to the ACL, PCL, lateral collateral ligament (LCL), medial collateral ligament (MCL), and PLC. OR reports, which all included fluoroscopic examination under anesthesia (EUA) and exploration at the time of ligamentous structure repair, were used as the gold standard. During fluoroscopic EUA, standard knee ligamentous tests, including the Lachman test, posterior drawer test, varus and valgus stress testing at both 0° and 30°, and the dial test, were consistently positive due to the complete ligamentous disruption inherent in all KDIV cases. An MRI report was considered accurate if it correctly identified all injuries documented in the OR report. If any injury described in the OR report was missed or mischaracterized in the MRI report, the case was classified as inaccurate. For example, if the MRI report described a sprain but the OR report confirmed a complete tear, the injury was considered misidentified by the MRI report. Postoperative follow-up notes were reviewed for functional outcomes, such as postoperative range of motion (ROM).

Statistical analyses were performed using SPSS 30.0 (IBM Corp., Armonk, NY, USA). Sensitivity, the proportion of correctly identified tears, was calculated individually for each ligamentous structure (ACL, PCL, MCL, LCL, and PLC). Because every patient sustained complete tears of all ligaments (Schenck KDIV pattern), the denominator (TP + FN) equals the total cohort size (*n* = 31) for each ligament, and the numerator (TP) equals the number of times the MRI reports correctly identified that tear. Specificity could not be determined, as no intact ligaments (true negatives) or false-positive MRI findings were present in this all-tear KDIV cohort. A one-way analysis of variance (ANOVA) was conducted to evaluate differences in misidentification rates among the ligaments, followed by a Tukey post hoc test to identify specific pairwise differences. The average range of motion (ROM) in flexion at final follow-up was calculated for the accurate and inaccurate cohorts, and an independent *t*-test was used to assess differences between the two groups.

## Results

A total of 31 patients (17 female, 14 male; average age 33.5 ± 11.3 years) met the inclusion criteria over the 10-year study period. Right-sided injuries occurred in 14 patients (45.2%) and left-sided injuries in 17 patients (54.8%). The predominant mechanism of injury was high-energy trauma in 18 patients (58.1%), followed by low-energy trauma in 12 patients (38.7%) and unknown mechanism in 1 patient (3.2%). Additional injuries were present in 18 patients (58.1%). The mean time from injury to surgery was 8.5 days ± 9.7 days (range 0–20 days), and the mean time from MRI to surgery was 6.6 days ± 4.3 days (range 0–14 days) (Table 2).

**Table 2:** Summary of patient demographics including age, sex, race, injury type, laterality, time from injury to surgery, time from MRI to surgery, mechanism of injury, and number of patients with additional injuries that did not involve the knee.

Patient demographics	
Total patients (*n*)	31
Age (Mean ± standard deviation)	33.5 years ± 11.3 years
Sex (*n*, %)	Female: 17 (54.8%), Male: 14 (45.2%)
Laterality	Right: 14 (45.2%), Left: 17 (54.8%)
Time from injury to surgery (Mean)	8.5 days ± 9.7 days (range: 0–20 Days)
Time from MRI to surgery (Mean)	6.6 days ± 4.3 days (range 0–14 days)
Mechanism of injury	High-energy: 18 (58.1%), Low-energy: 12 (38.7%), Unknown: 1 (3.2%)
Patients with additional Injuries	18/31 (58.1%)

**Figure 2:**
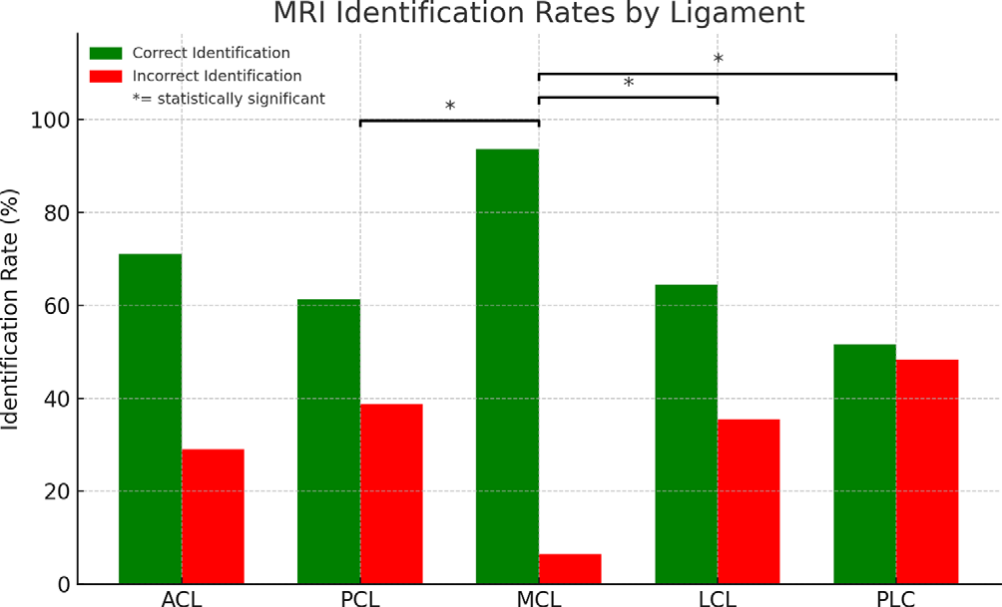
Sensitivity (Correct identification rates) and misidentification rates of ligamentous injuries on MRI. Asterisks denote statistically significant differences in misidentification rates between the MCL and the LCL, PCL, and PLC based on a one-way ANOVA followed by a Tukey post hoc test (*p* < 0.05).

**Figure 3:**
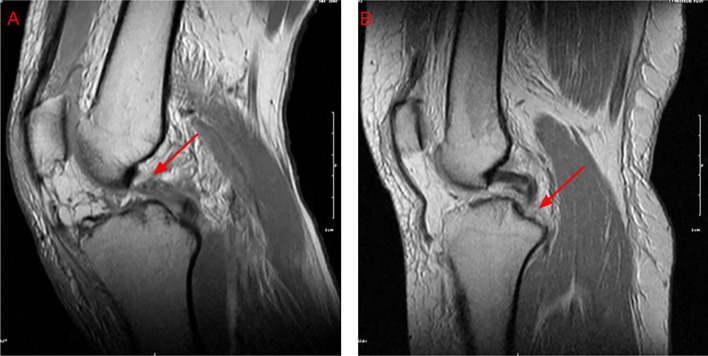
Examples of posterior cruciate ligament (PCL) injuries on pre-operative MRI. (A) Sagittal proton-density fat-saturated image from a case correctly reported as a full-thickness PCL tear (arrow), showing complete discontinuity of the ligament fibers. (B) Sagittal proton-density fat-saturated image from a case in which a displaced tibial-side avulsion (arrowhead) was mis-reported as an intact PCL, illustrating a false-negative interpretation.

**Figure 4:**
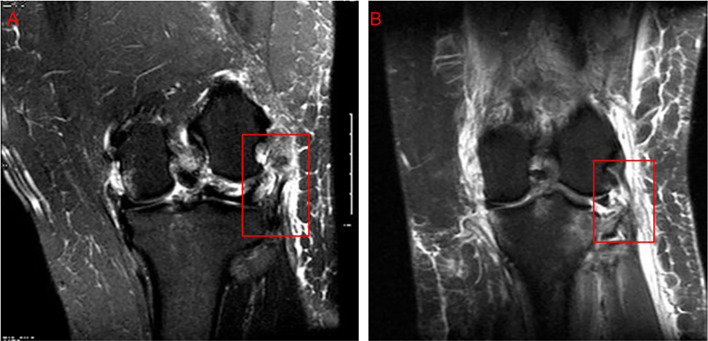
Examples of lateral collateral ligament (LCL) injuries on pre-operative MRI. (A) Coronal fat-suppressed proton‐density image from a case correctly reported as a full-thickness LCL tear (box), showing complete discontinuity of the ligament fibers. (B) Coronal fat-suppressed proton‐density image from a case in which a midsubstance LCL tear (box) was mis-reported as an intact ligament, illustrating a false-negative interpretation.

**Figure 5:**
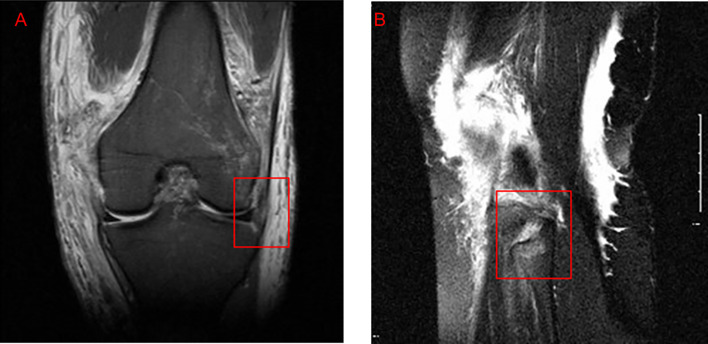
Examples of posterolateral corner (PLC) injuries that were misinterpreted as intact on pre-operative MRI. (A) Coronal fat-suppressed proton-density image shows a full-thickness tear of the iliotibial band (box), reported as intact in the original radiology report. (B) Sagittal proton-density image from a different case that demonstrates disinsertion of the biceps femoris tendon with bone marrow edema of the fibular head (box), also misreported as intact.

The MRI reports were analyzed by eight different fellowship-trained musculoskeletal radiologists over the 10-year study period. 16 (51.6%) of the reports were interpreted by the same radiologist, and the remaining 15 (48.4%) were evaluated by the other seven radiologists. Overall sensitivity (correct identification rate) differed markedly by ligament. MRI detected ACL tears in 22 of 31 cases (71.0%), PCL tears in 19 of 31 (61.3%), and LCL tears in 20 of 31 (64.5%). Detection was poorest for PLC injuries, with only 16 of 31 tears identified (51.6%), and highest for the MCL, where 29 of 31 cases (93.5%) were detected (Figure 2). Additionally, only 11 out of 31 (35.5%) MRI reports fully aligned with intraoperative findings, highlighting significant variability in MRI accuracy. Representative MRI images demonstrating misreported ligamentous injuries to the PCL, LCL, and PLC are shown in Figures 3–5.

A one-way ANOVA revealed a statistically significant difference in sensitivity across ligament types (*F*(4, 150) = 4.529; *p* = 0.0018). Post hoc analysis using a Tukey test showed that the sensitivity of PLC injuries was significantly lower than the sensitivity of MCL injuries (*p* = 0.0033). Furthermore, the LCL (*p* = 0.047) and PCL (*p* = 0.044) also had significantly lower sensitivities than the MCL. No other significant differences between ligament misidentification rates were seen in the post hoc Tukey test.

Follow-up data were available for 28 of 31 patients (minimum 6 months), with 2 patients lost to follow-up in the accurate-MRI cohort and 1 in the inaccurate MRI cohort (*n* = 9 and *n* = 19, respectively). The average length of follow-up was 35.56 ± 19.34 months (range: 6–136 months) in the accurate MRI group and 32.71 ± 20.53 months (range: 6–153 months) in the inaccurate group. The mean ROM at final follow-up was 95.0 ± 20.62° (range: 60–120) in the accurate cohort and 88.95 ± 26.64° (range: 35–120) in the inaccurate cohort. This difference in ROM was not statistically significant (*p* = 0.56).

## Discussion

This study reveals significant diagnostic limitations in MRI radiology reports for detecting ACL, PCL, LCL, and PLC injuries in KDIV knee dislocations. The MRI reports were relatively accurate for MCL injuries, which is consistent with prior findings that MRI studies of the MCL have a 65–92% correlation with clinical findings [9]. However, the accuracy dropped substantially for PLC injuries, which were missed in nearly half of the cases. A one-way ANOVA confirmed these discrepancies, and a post hoc Tukey test revealed that PLC, PCL, and LCL injuries were all significantly more likely to be misidentified than MCL injuries. No other pairwise differences reached statistical significance. These statistical results support this study’s observation that MRI is more reliable for medial-sided injuries and less dependable for diagnosing complex lateral and posterior structures, particularly the PLC. Overall, our findings suggest that MRI reports should be interpreted with caution and integrated with fluoroscopic EUA at the time of surgery to provide the most accurate and comprehensive assessment of ligamentous injury.

Several previous studies have investigated the diagnostic utility of MRI for ligamentous injuries in knee dislocations under controlled conditions where the MRI images were retrospectively reevaluated [6–7, 10]. One study of 39 patients who underwent PLC repair reported MRI sensitivities of 95% for the LCL and 82% for the popliteus tendon when images were reinterpreted by trained musculoskeletal radiologists after surgical intervention [10]. Other studies have similarly rereviewed MRI images and concluded that MRI is generally reliable for identifying cruciate and collateral ligament injuries but consistently less dependable in detecting PLC injuries [6, 7]. For example, a larger study of 97 heterogeneous injuries found that MRI findings were consistent with intraoperative results in only 52.6% of cases (51 patients), with just 11% of PLC tears correctly identified [7]. That study concluded that MRI was largely uninformative in complex knee dislocations, including 26 cases with both PLC and LCL injuries [7]. In contrast, a separate multicenter study that evaluated existing MRI radiology reports of 178 patients with multiligament knee injuries reported high sensitivities for the ACL (96.8%) and PCL (93.6%) and lower sensitivities for the LCL (79.8%) and popliteus tendon (71.2%) [11]. However, that study included a heterogeneous injury pattern, with knee dislocations ranging from KDI to KDIV, and did not focus on the real-time diagnostic value of MRI reports [11].

By analyzing the MRI reports available at the time of care and not reinterpreting the MRI images, our findings more accurately reflect clinical realities, where surgeons rely on the radiology report issued at the time of care for preoperative planning [8]. Furthermore, by limiting our analysis to confirmed KDIV knee dislocations, we minimized variability, maintained consistency, and focused on a clinically significant and severe injury pattern. As a result, this study makes a unique contribution to the existing body of literature by shifting the focus away from controlled, retrospective MRI reinterpretation and toward real-world diagnostic performance in a standardized injury pattern. Prior studies have assessed MRI accuracy through retrospective image re-reviews by radiologists after operative intervention [6–7, 10], which can introduce interpretation bias and potentially inflate the perceived accuracy of MRI. In contrast, our methodology reflects the actual diagnostic information available during clinical decision-making. This distinction emphasizes the variability and limitations of MRI reports in complex knee trauma and underscores the need for improved imaging interpretation in real-time clinical settings.

The gap between theoretical diagnostic accuracy and practical utility warrants careful consideration. In our cohort, functional recovery did not differ between the accurate and inaccurate MRI cohorts. With a minimum of 6 months follow-up, mean flexion ROM was 95.0 ± 20.62° in the accurate MRI group (*n* = 9) versus 88.95 ± 26.64° in the inaccurate group (*n* = 19) (*p* = 0.56). We believe these equivalent outcomes reflect the routine use of fluoroscopic examination under anesthesia at the time of surgery, which allowed precise identification of all torn ligaments and comprehensive reconstruction regardless of preoperative MRI accuracy. Thus, even when MRI misidentification occurred, intraoperative fluoroscopic EUA acted as a safeguard against missed injuries and helped ensure similar postoperative function in both cohorts.

Limitations of this study include the relatively small sample size of 31 patients and the retrospective design of the study. While the findings highlight important trends in real-world MRI accuracy for KDIV knee dislocations, a larger cohort would allow for more definitive conclusions and greater statistical power. Additionally, the use of radiology reports dictated by eight different radiologists introduces heterogeneity in interpretation, though this also reflects real-world practice. To minimize variability, we limited our analysis to KDIV cases, allowing for a more consistent evaluation of MRI performance in a specific complex injury pattern. Future directions include a secondary, blinded radiological review of the MRI images to determine whether discrepancies between radiology reports and intraoperative findings stem from interpretive error or inherent MRI imaging limitations. This approach may clarify the reasons behind missed ligamentous injuries and help guide strategies to enhance diagnostic accuracy.

In conclusion, in patients with KDIV knee dislocations, preoperative MRI radiology reports demonstrated substantial limitations in accurately identifying ligamentous damage, particularly to the PCL, LCL, and PLC. While MCL injuries were reliably reported, only 35.5% of MRI reports fully matched intraoperative findings. To achieve the most accurate assessment of ligamentous damage, MRI findings should be interpreted alongside fluoroscopic EUA at the time of surgery.

## Data Availability

The data supporting the findings of this study are available from the corresponding author upon reasonable request.
